# Cyanobacterial Cyclopeptides as Lead Compounds to Novel Targeted Cancer Drugs

**DOI:** 10.3390/md8030629

**Published:** 2010-03-15

**Authors:** Ioannis Sainis, Demosthenes Fokas, Katerina Vareli, Andreas G. Tzakos, Valentinos Kounnis, Evangelos Briasoulis

**Affiliations:** 1 Human Cancer Biobank Center, University of Ioannina, Greece; E-Mails: isainis@cc.uoi.gr (I.S.); kvareli@cc.uoi.gr (K.V.); atzakos@cc.uoi.gr (A.T.); 2 Department of Materials Science and Engineering, University of Ioannina, Greece; E-Mail: dfokas@cc.uoi.gr (D.F.); 3 Department of Biological Applications and Technologies, University of Ioannina, Greece; 4 Department of Chemistry, University of Ioannina, Greece; 5 School of Medicine, University of Ioannina, Greece; E-Mail: vkounnis@cc.uoi.gr (V.K.)

**Keywords:** microcystin, cyanobacteria, cyanotoxins, cancer, targeted-therapy, OATP, membrane transporters

## Abstract

Cyanobacterial cyclopeptides, including microcystins and nodularins, are considered a health hazard to humans due to the possible toxic effects of high consumption. From a pharmacological standpoint, microcystins are stable hydrophilic cyclic heptapeptides with a potential to cause cellular damage following uptake *via* organic anion-transporting polypeptides (OATP). Their intracellular biological effects involve inhibition of catalytic subunits of protein phosphatase 1 (PP1) and PP2, glutathione depletion and generation of reactive oxygen species (ROS). Interestingly, certain OATPs are prominently expressed in cancers as compared to normal tissues, qualifying MC as potential candidates for cancer drug development. In the era of targeted cancer therapy, cyanotoxins comprise a rich source of natural cytotoxic compounds with a potential to target cancers expressing specific uptake transporters. Moreover, their structure offers opportunities for combinatorial engineering to enhance the therapeutic index and resolve organ-specific toxicity issues. In this article, we revisit cyanobacterial cyclopeptides as potential novel targets for anticancer drugs by summarizing existing biomedical evidence, presenting structure-activity data and discussing developmental perspectives.

## 1. Introduction

**Figure 1:**
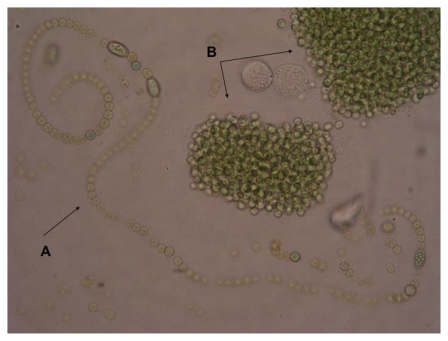
Typical colony-forming cyanobacteria found in a toxic bloom in a Mediterranean lake (Lake Pamvotis Greece). A. *Anabaena* sp. B. *Microcystis* sp.

Cyanobacteria (blue-green algae) appeared approximately 3.5 billion years ago, triggering major ecological change through photochemical release of molecular oxygen from water into the atmosphere [1,2]. The cyanobacteria population comprises 150 genera and about 2000 species of considerable diversity. They are prokaryotic algae that exist as unicellular species or in colonies (Figure 1). Due to their photosynthetic capacity, they constitute the primary first level organisms in food chains in water ecosystems. Moreover, these prokaryotes play a significant role in the marine nitrogen cycle and also have a role in balancing nitrogen (N) and CO_2_ dynamics in the biosphere [3]. Besides their life-sustaining role, the eutrophic growth of certain species of cyanobacteria in water reservoirs (toxic cyanobacterial water-blooms) have generated increasing concerns for human and animal health due to the detrimental effects of the toxins they produce [4–7]. Therefore, regular monitoring of suburban water reservoirs for cyanotoxins has become a necessity and is encouraged [8,9]. In the case of humans, the prime cyanobacterial toxicoses are acute liver damage, neurotoxicity, gastrointestinal disturbances and liver cancer, all of which demonstrate the potent biological activity of cyanotoxins [10,11]. However, from a pharmacological point of view, the targeted biological activities and characteristic physicochemical properties of cyanotoxins identify these molecules as potent candidates that warrant pharmacological exploitation as targeted cancer therapeutics. More specifically, cyanobacterial cyclopeptides share a pharmacophore structure that may lead to development of a novel class of anticancer therapeutics with activity against chemotherapy-refractory metastatic cancers that express organic anion transporters, the primary molecular targets of these compounds [12,13]. In this article, we focus on microcystin (MC) as a potential anticancer compound and present relevant supporting data.

## 2. Cyanotoxins–Microcystin

### 2.1. Categories

Cyanobacterial toxins (cyanotoxins) belong to diverse chemical classes and can cause cell-specific toxicity such as neurotoxicity by anatoxin-a, anatoxin-a(S) and saxitoxins; hepatotoxicity by microcystins, nodularin and cylindrospermopsin; and dermatitis by lyngbyatoxin-a [14]. Among cyanotoxins, the cyclic cyanotoxins nodularins and MC are among the most common natural toxins. They have been well studied and have been shown to share similar mechanisms of biochemical action. Both are potent inhibitors of the serine/threonine protein phosphatase families PP1 and PP2A and also pro-oxidants with a potency to induce cell damaging oxidative stress through generation of reactive oxygen species (ROS) [15,16].

### 2.2. Microcystins: Physicochemical Characteristics

**Figure 2:**
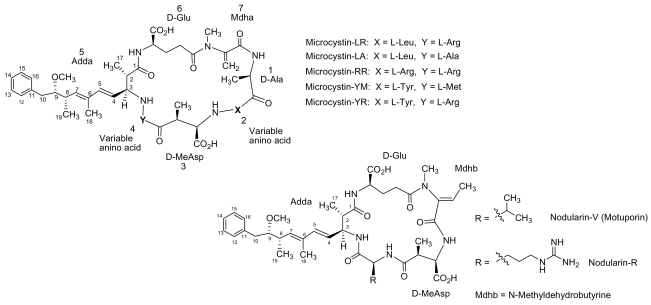
Structures of the most common isolated MCs and nodularins.

Microcystins (MC) are cyclic heptapeptides with a relative molecular mass (M_r_) varying between 500 and 4000 Da. They were first isolated from a strain of *Microcystis aeruginosa* and named after this organism. High MC content has also been found in other species such as *Anabaena, Planktothrix, Nostoc, Anabaenopsis, Aphanocapsa* and in the soil cyanobacterium *Haphalosiphon*. The MCs comprise a series of more than 60 cyclic heptapeptides with the general structure *cyclo*-(D-Ala-X-(D)-*erythro*-*β*-methyl-*iso*-Asp-Y-Adda-(D)-*iso*-Glu-*N*-methyldehydro-Ala-). The amino acid residues are numbered sequentially from (D)-alanine (1) to *N*-methyldehydroalanine (7), while the letters X and Y represent variable positions that are occupied by natural L-amino acids in the molecule (Figure 2) [17–19].

Among MC variants, the most toxic and common is MC-LR, in which the two variable amino acids are leucine and arginine [20]. The presence of the amino acid, Adda ([2*S*,3*S*,8*S*,9*S*]-3-amino-9-methoxy-2,6,8-trimethyl-10-phenyldeca-4,6-dienoic acid), is an unusual and specific structural feature of MCs [21]. Adda plays an important role in the biological activity of MC since as hydrogenation or ozonolysis of the diene system in this unit results in an inactive product [22,23]. Moreover, acylation of glutamate renders MCs less toxic or even nontoxic [24,25].

From a pharmacological standpoint MC-LR possesses several desirable properties such as water solubility and extreme stability in several exposure and handling conditions. Their stability in reservoir water is less than one week, but they are stable for longer periods of time in filtered or deionized water [26,27]. Moreover, MC-LR remains stable even after several hours of boiling, and it is also resistant to chemical hydrolysis or oxidation at near-neutral pH [28–30].

### 2.3. Microcystin Biogenesis and Ecological Role and Function

Microcystins are synthesized by a nonribosomal enzyme complex, as are most cyanobacterial peptides, encoded by the microcystin (*mcy*) gene cluster. *Mcy* spans ~55 kb and includes genes for peptide synthetases, polyketide synthases, mixed peptide synthetases and tailoring enzymes [31]. Their ecological role and function is currently unresolved although it is clear they have numerous effects on phytoplankton and zooplankton [32,33]. It has been proposed that MCs have evolved to function as a defense mechanism of cyanobacteria against grazing, a theory that has been debated by recent findings indicating that microcystin synthetase predated the metazoan lineage [34,35]. Other investigators have also found that MC is produced in response to extracellular metabolites released by herbivorous zooplankton [36,37] and that they may scavenge environmental metals, such as iron [38].

### 2.4. Biological Activity of Microcystins as Xenobiotics

#### 2.4.1. In Animals

##### 2.4.1.1. Acute Exposure

The first report of lethal intoxication of animals that drank water with a high burden of algal blooms was reported in Australia 140 years ago [4]. It later became clear that acute exposure to MCs can cause severe hepatocellular damage in animals and thus MCs were named hepatotoxins [39,40]. Considerable variation among animals is observed with regard to MC toxic sensitivity [41]. In mice the oral lethal dose that kills 50% of subjects (LD50) for MC-LR ranges from 5 to 10 mg/Kg bodyweight (bw), compared to 0.1 mg/kg bw in rats [42,43]. The intraperitoneal LD50 of MC-LR in mice and rats also varies, but a value of 50–100 μg/gr bw is commonly accepted [11,44]. With regard to other MCs the i.p LD50 for MC–LA, -YR, -YM are similar to that of MC-LR, but the i.p LD50 for MC-RR is ten times higher than that of MC-LR [5].

##### 2.4.1.2. Low-dose Chronic Exposure

Chronic exposure of experimental animals to sub-lethal low doses of MC has been shown to promote tumorigenesis in coordination with dysfunctional *p53* [45]. Moreover, in two-stage carcinogenicity assays, chronic MC exposure was shown to promote liver tumorigenesis once initiation occurred with known carcinogens diethylnitrosamine [46] azoxymethane [47] and particularly with aflatoxin B1 [48,49].

#### 2.4.2. In Humans

##### 2.4.2.1. Acute Exposure

The potential of MC to induce lethal toxicity in humans was recently recognized following a biological accident at a dialysis center in Caruaru, Brazil in 1996. A total of 100 out of 131 hemodialysis patients developed acute liver failure, and 76 died following accidental intravenous exposure to MC that had contaminated the dialysis water source (a municipal water supply). It was estimated that 19.5 μg/L MC was in the water used for dialysis and the concentration of MC found in liver tissue from patients who died ranged from 0.03 to 0.60 mg per kilogram of liver tissue (median, 0.18) [50–52]. Another minor incident of MC exposure in hemodialysis patients was also reported a few years later in Rio de Janeiro, Brazil. In this case, serum MC concentrations in patients ranged from <0.16 to 0.96 ng/mL, and no fatalities occurred [53].

##### 2.4.2.2. Low-dose Chronic Exposure

Data on chronic low-dose exposure to MCs are limited and mainly originate from epidemiological studies. A correlation between the high incidence of primary liver cancer and drinking water contaminated with MC during the summer time was first observed in certain provinces in China [54]. Similarly, an increased incidence of primary liver cancer was recorded during the last decade in certain Serbian regions in which the citizens made use of blooming water reservoirs for drinking purposes [55]. These epidemiology data, supported by *in vivo* studies, indicate that a combined exposure to hepatocarcinogen aflatoxin B1, hepatitis B virus and an intermittent intake of MCs may drive liver carcinogenesis [49,54]. In response to these observations and studies on MC toxicity, the WHO issued a provisional guideline for drinking water and set 1 μg/L MC-LR concentration as the upper cut-off safe level for drinking water [11,56].

## 3. MC-LR Cell Molecular Targets

**Figure 3:**
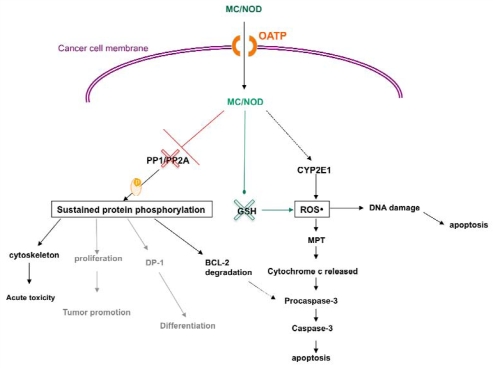
Cancer cell molecular targets and actions of cyanobacterial cyclopeptides. (MC = microcystin, NOD = nodularin, OATP = Organic Anion Transporting Polypeptides, PP = protein phosphatase, MPT = Mitochondrial permeability transition, ROS = reactive oxygen species, GSH = glutathione, CYP2E1 = Cytochrome P450 2E1, red cross symbol = inhibition, green cross symbol = consumption, black lines symbolize established action, grey lines symbolize likely actions).

The hydrophilic structure of most MC variants hinders their penetration through plasma cell membranes and therefore cell uptake is facilitated by a transporting system. Such transporters dedicated to handle intracellular transport of cyclic peptides are the organic anion transporting polypeptides (OATP) [57,58]. Currently, there is limited data on the transport of the various MC analogues by different members of the OATP family. According to our knowledge, MC variants and especially the most studied MC-LR has been identified as substrate for OATP1A2, OATP1B1 and OATP1B3 [58]. In mammals consuming water or food contaminated with MCs, the toxins are transported through the small intestine to the bloodstream *via* OATP-expressing epithelial cells that line the small intestine. *Via* the bloodstream, MCs finally concentrate in hepatocytes, which overexpress OATP. The hepatocellular toxic effects are generally attributed to disruption of reversible protein phosphorylation dynamics though inhibition of protein phosphatases (PP) type 1 and type 2A and the uncontrolled generation of high levels of ROS [59–62] (Figure 3).

It has been reported that MC-LR covalently binds to cysteine residues of PP1 and PP2A [63]. As a result, inhibition of PP1 and PP2A leads to hyperphosphorylation of functional and cytoskeletal proteins and finally to cell apoptosis [39,64]. Macroscopically, the liver becomes swollen and hemorrhagic and the dissociation and disruption of the liver epithelium is extensive [65–67]. It is interesting that MC-RR, while an equally potent inhibitor of PP as MC-LR, requires higher concentrations to induce both hepatocyte deformation and increased protein phosphorylation. The reduced hepatotoxicity of MC-RR likely reflects a reduced affinity for the transporter [68]. In chronic low-dose exposure, MCs are also thought to exert tumor-promoting effects through inhibition of PP1 and PP2A, which are known to function as tumor suppressors [69]. PP inhibition leads in a shift in the balance between phosphorylation/dephosphorylation towards a higher phosphorylation status of target genes. For instance, PP2A has been shown to regulate the activity of at least 50 protein kinases, and thus the effect of a potent inhibitor of PP2A activity is likely to be detrimental to the cell. Protein kinase C (PKC), Akt, extracellular signal-regulate kinase (ERK), mitogen-activated protein kinase (MAPK), IκB kinase, p38 and caspase-3 are some of the proteins regulated by PP2A [46,70–72].

MC has also been shown to exert genotoxic effects such as DNA fragmentation, chromosomal aberrations, micronuclei formation, loss of heterozygosity and even base substitution mutations [10,73–75]. ROS are known to damage DNA. In this context, cyanobacterial cyclopeptides have been reported to produce free radicals and alter intracellular reduced glutathione (GSH) [76,77]. Moreover, i.p. treatment of mice with nodularin decreased the enzymatic activity of superoxide dismutase, catalase, and glutathione peroxidase [78].

Microcystins can also affect intracellular targets other than PP2A. For instance, the h subunit of ATP-synthase was shown to be a MC binding protein and thus a possible target in liver cells [79]. Another potential target of MC could be mitochondrial aldehyde dehydrogenase II, which has been recently identified as a MC target in the human liver [80]. Given the important role of aldehyde dehydrogenase II in acetaldehyde detoxification and the prevention of free radical formation, the physical association of this enzyme with MC may, to some extent, explain the hepatotoxicity of MCs.

## 4. Cytotoxic Effects of MC-LR

### 4.1. Activity of MC in Normal Cell Lines and Tissues

In primary cultured rat hepatocytes treated with MC-LR for 24 and 72 h, LC50 values were found to be 48 ng/mL and 8 ng/mL, respectively. Exposure of rat hepatocytes to sub-lethal concentrations of MC-LR of 2 or 10 ng/mL for 3, 24 or 48 h results in the formation of ROS as demonstrated by an acute increase in intracellular reduced glutathione [16]. Extracts containing high concentrations of MC (675–2700 nM MC-LR eq.) caused apoptotic effects in rat hepatocytes and human lymphocytes. In rat hepatocytes morphological apoptotic changes were first observed after 30 min of incubation, but characteristic biochemical changes were not seen. In human lymphocytes, apoptotic morphological changes were seen 24 h after incubation. An incubation period of 48 h was judged optimal for the appearance of internucleosomal DNA degradation [81]. ROS were found to play a critical role in the mitochondrial permeability transition (MPT) after treatment of rat hepatocytes with MC-LR [82].

Ultra-rapid apoptosis was observed in primary hepatocytes following microinjection with both MC-LR and nodularin, characterized by cytoplasmic shrinkage, chromatin condensation, membrane blebbing, and procaspase-3 cleavage [83]. The finding that PP2A regulates BCL-2 phosphorylation is obviously of major therapeutic importance. Lin *et al.* found PP2A inhibition results in proteasome-mediated degradation of the hyperphosphorylated BCL-2 at the endoplasmic reticulum [84]. Moreover, pharmacologic inhibition or RNA interference knockout of PP2A caused proteasomic degradation of phosphorylated BCL-2 and sensitized the cells to various cell death stimuli [85]. Taken together, these findings imply that MC can promote apoptosis in cancer cells that overexpress BCL-2 *via* a PP2A inhibition approach. ROS generation and resultant DNA damage were associated with MC-induced toxicity in other non-malignant non-liver cells [86,87].

### 4.2. Activity of MC in Cancer Cells

To consider MCs as targets to developing potent anticancer drugs, tumor cells should demonstrate selective sensitivity to MC. Unfortunately the investigation of anticancer effects of MCs *in vitro* has been difficult due to the down-regulation of transporters in most cancer cell lines. This lack of expression agreement between transformed and ortholog cell lines and the corresponding tissue is a known problem with cell transporters attributed to down-regulation of transporter genes when cells are maintained in culture [88,89]. Characteristically, in freshly-isolated trout and murine hepatocytes a rapid loss of OATP transporter gene expression was observed and coincided with a loss of MC sensitivity in these cultured cells [90].

On the contrary, there is clear evidence that OATP are over-expressed in cancer tissues (*detailed data are presented in the following section*) [91]. Characteristically, OATP1B1 and OATP1B3 expression assessed by western blot assay was found in hepatocellular carcinoma [92]. In addition, OATP1B1 and OATP1B3 expression was detected in a few cell lines originating from liver, colon, and pancreatic tumors [93].

Zegura *et al.* have found that MC-LR induced DNA damage in HepG2 hepatoma cells related to decreased intracellular glutathione [77]. In another study, they observed a significantly increased ratio of expression of bax to bcl-2 induced by MC-LR, which suggests that apoptosis in HepG2 cells proceeds *via* the mitochondrial pathway [73]. Moreover, Monks at el., following observations that OATP1B3 mRNA is up-regulated in non-small cell lung cancer, transfected HeLa cervical cancer cells with the drug transporters OATP1B1 and OATP1B3 to create in vitro models in which MCs could gain intracellular access and test the activity of MCs against OATP-expressing cancer cells. Transfected HeLa cells were found to be 1,000-fold more sensitive to MC-LR than the vector-transfected control cells, showing that transporter expression imparts marked selectivity for MC cytotoxicity [12]. This finding suggests that MC cytotoxicity in OATP1B1- and OATP1B3-expressing HeLa cells is related to cell-specific inhibition of PP2A and not to PP inhibition in general.

## 5. Organic Anion Transporting Polypeptides

According to the Human Genome Organisation Gene Nomenclature Committee (http://www.genenames.org) the *SLCO* acronym is used for naming genes and OATP for the corresponding protein product of each gene. The gene official symbol SLCO stands for Solute Carrier Organic anion transporter family. The superfamily of OATP is further divided into families and subfamilies according to their amino acid sequence identity, ≥40% and ≥60%, respectively. The OATP families include six members that are symbolized by OATP1, OATP2, OATP3, OATP4, OATP5 and OATP6. Next to the Arabic numeric system, which signifies the OATP family, a Latin letter is used to specify the subfamily type, *i.e.*, OATP1A. Further classification of each subfamily member is achieved with an Arabic number next to the subfamily letter that corresponds to the order of discovery of the gene/product of each member.

### 5.1. OATP Substrates

Apart from MCs, numerous other endo- and xenobiotics with diverse chemical structures have been identified as substrates for members of the OATP family. Among them are hormones (mainly steroid and thyroid hormones), eicosanoids, bilirubin, bile acids, and drugs such as HMG-CoA-reductase inhibitors (holesterol lowering statins), digoxin, angiotensin-II receptor antagonists like olmesartan, antibiotics like levofloxacin and anticancer agents like methotrexate and taxanes. Each OATP subtype presents different substrate specificities. A comprehensive review of endogenous and xenobiotic substrates for human OATP family members, was recently published by König *et al.* [94].

### 5.2. OATP Expression in Normal Human Tissues

Depending on the family and subfamily type, some OATP members (Table 1) show a very specific profile of expression, *i.e.*, 1B1 and 1B3 are both found in the liver [58,92,95,96] and mononuclear cells [96], while expression of *SLCO1B3* mRNA was detected in the cervix [96]. OATP6A1, also known as the gonad-specific anion transporter, is mainly identified in the testis [97,98], but also in the spleen, brain, fetal brain and the placenta [98] (Table 1).

Other OATP members show a less specific expression profile. OATP1A2 is found in the liver [95,99–101], the brain [58,95,96,100,102,103], the kidney [104], the eye [100], the prostate [95], and the mammary gland [105]. OATP1C1 is mainly located in the brain, testis, heart, eye and mammary gland [96,100,103,105]. OATP4C1 is identified in the kidney [103,106], and also in the lungs, skin, white blood cells (neutrophils, mononuclear cells and peripheral leukocytes), mammary gland and liver [96,107]. OATP5A1 is identified mainly in the prostate, skeletal muscles, thymus [96], on classically activated macrophages [108], and breast [107]. Other OATP members such as 2A1, 2B1, 3A1 and 4A1 are distributed ubiquitously throughout the human body [96,103,109–115]. Hence the brain and the liver appear to be the tissues with the most prominent OATP expression, along with the testes, breasts and kidneys.

### 5.3. OATP Expression in Human Cancers

OATP expression in cancers has been poorly investigated (Table 1). Nevertheless, among OATP members OATP1B1 and 1B3 were found expressed mainly in hepatocellular carcinoma [92,116,117] with the second (1B3) also expressed in colon cancer [91], breast cancer [118], and non-small cell lung cancer [12]. OATP1A2, 1C1, 2B1 and 4A1 show an expression preference for gliomas [102] and primary or metastatic bone tumors [119]. In addition, OATP2B1 and 4A1 appear to also be expressed in breast, colon and lung cancers [107,119]. Similarly, OATP 2A1, 3A1, 4C1 and 5A1 show a common expression profile for breast cancer and bone tumors [107,119]. Furthermore, 2A1, 3A1 and 4C1 expression has been identified in lung cancer, and 2A1 and 3A1 in colon cancer [96,107]. The gonad-specific transporter OATP6A1 has shown a rather high-frequency expression in non-small cell lung cancer, bladder tumors, esophageal tumors and in medulloblastoma [98,120].

We consider that the mechanisms that modulate OATP expression in cancer tissues warrant rigorous investigation. Obviously more studies are needed to map OATP expression in human cancers as the target of an emerging novel class of targeted therapeutics for the treatment of refractory metastatic cancers.

### 5.4. OATP: Cancer Trapdoors to Be Exploited

Despite small incremental improvements achieved with the introduction of targeted cancer therapeutics in clinical practice, it is widely accepted that systemic therapy of several advanced common cancers has failed to meet hopeful expectations and unfortunately remains an unmet need [121–123]. It is now accepted that a kernel of refractory stem-like cancer cells turn these tumors into “hard to die” cancers [124,125]. To address this therapeutic limitation, oncological research has shifted its focus to individualized therapeutic approaches with the aim of maximizing treatment benefits [126]. However, novel therapeutic strategies, more specific targets and innovative therapies are desperately needed to improve therapeutic options [127,128]. In this area, the influx membrane transporters of selective substrates appear to be an overlooked option, although they have been clinically proven to be a valid therapeutic target [129,130].

With regard to MC, the low growth inhibition activity of MC-LR (GI50 > 5 μM at 24 h) in cancer cell lines [131] due to its low cell permeability in the absence of specific transporters, in conjunction with the moderate selectivity profile for PP1 and PP2A inhibition, has led the scientific community to overlook their therapeutic potential for years [132]. Interest is now revived following elucidation of knowledge on cell influx transporters of MC.

However, to achieve a clinically meaningful therapeutic window for MC-derived therapeutics, the issue of anticipated hepatic toxicity of these agents needs to be addressed. It is promising that in OATP-transfected HeLa cells, MC induced cytotoxic effects at concentrations in the subnanomolar range, significantly lower than the doses required for hepatic toxicity in experimental animals [12]. This finding suggests that normal hepatocytes may differ from cancer cells in sensitivity to MC for metabolic reasons.

Such metabolic differences between normal and malignant cells in the context of MC activity have been explored. Microcystin and the related toxic cyclic peptide nodularin are shown to stimulate glutathione-dependent detoxification pathways in normal hepatocytes. Exposure of rat hepatocytes to sub-lethal concentrations of MC-LR resulted in an acute increase in intracellular glutathione in parallel with increased ROS [16]. Addition of *N*-acetylcysteine (NAC) to the culture medium, an agent that increases intracellular glutathione concentrations, decreased sensitivity of cultured rat hepatocytes to MC. Conversely, cultured hepatocytes treated with buthionine sulfoximine, an agent that decreases intracellular glutathione, became increasingly sensitive to cyanobacterial extract [82]. These studies suggest that glutathione plays a role in the *in vivo* hepatic detoxification of MCs. In contrast, in the HeLa cell model neither NAC nor buthionine sulfoximine affected MC toxicity, indicating that there are metabolic differences in MC intracellular targets between hepatocytes and cancer cells [12]. Finally, based on the tissue distribution, excretion and hepatic biotransformation of MC-LR, toxicity is considered to be related to long-term cellular retention, presumably through covalent binding of the toxin or its metabolites with high molecular weight components [133].

## 6. MC Analogues: Potentials and Perspectives

It has been suggested that developing MC-LR structural analogs of higher cancer specificity and selected for a broader therapeutic index may efficiently target OATP-expressing tumors [134]. This perspective is well served by the chemical structure of these natural cytotoxins as shown below.

### 6.1. Combinatorial Chemical Synthesis

#### 6.1.1. The Adda Issue

SAR data revealed that the Adda and D-glutamic acid regions play highly important roles in the hepatotoxicity of MCs, providing a steric configuration that is directly involved both in the carrier protein, conveying cell specificity as well as at the active site of protein phosphatase [135]. The crystal structure of mammalian PP1 complexed with MC-LR confirmed the major aspects of the conserved acid binding domain pharmacophore model [136]. The glutamic acid carboxyl group and the Adda carbonyl group bind to the metal-binding site *via* metal-liganded water. In addition, the MeAsp carboxyl group hydrogen binds to Arg 96 and Tyr 134. The long hydrophobic tail of Adda is placed in the hydrophobic groove region of PP1, adjacent to the active site, while the L-arginine side chain is fully exposed to the solvent and does not form any significant contacts with PP1 or PP2A. The carbon of the *N*-methyldehydroalanine side-chain is covalently linked to the Cys 273 of PP1, a linkage that is secondary to the inhibition activity of the toxin and likely occurs as a delayed reaction in solution [136,137]. However, the biological relevance of this reaction is unknown. Although they share similar biological properties, MCs and nodularins have important functional differences with respect to their interaction with PP1 and PP2A. Although both toxins initially bind non-covalently and inhibit these enzymes, crystallographic data shows that nodularins, including motuporin, do not bind covalently to PP1 or PP2A even as a delayed reaction [138]. This fact is despite the presence of an *N*-methyldehydrobutyrine (NMdhb) residue that could undergo a Michael addition reaction with Cys 273, similar to the reaction of the Nmdha residue in MC-LR.

Due to their unique biological properties, MCs and nodularins have been the target of synthetic organic chemists in the pursuit of a total synthesis that can provide access to a series of structurally modified analogues. This work resulted in the total synthesis of MC-LA (MC-LA) [139]. In an effort to design compounds with increased PP1 selectivity, Chamberlin synthesized new MC-LA variants (Table 2) inspired by his total synthesis of MC-LA [139]. Replacement of the L-leucine residue with cyclohexylalanine led to a variant of MC-LA with a seven-fold increased selectivity for PP1 and high potency (0.52 nm) [140]. Despite its high potency and the best selectivity profile reported to date, the low cell-permeability of this cyclic inhibitor would limit its further development. Also, a series of greatly simplified analogues comprised only of Adda, and of a single additional amino acid, or of synthetic linearized and truncated peptides, were prepared, a few of which retained moderate activity as PP1/PP2A inhibitors [141]. On the contrary, linear analogs of microcystins MC-RR, MC-YM, MC-LA in which the Adda region was neither incorporated nor replaced by another hydrophobic residue were inactive, and did not reveal any signs of toxicity in white mice [142,143]. Moreover, an Adda-based compound, a mimic of the RVXF peptides, was found to bind at the PP1 regulatory site and activate the enzyme, in direct contrast to its strongly inhibiting progenitor MC-LR [144].

#### 6.1.2. Synthetic Approaches

We are in the process of harnessing the power of synthetic organic chemistry to attenuate the toxicity of MCs by designing structural variants with an improved therapeutic profile. Entry to such MC analogues can be achieved by a) the direct chemical elaboration of the MC scaffold exploiting the dense array of functional groups and/or b) the *de novo* design and synthesis of new MC variants exploiting the synthetic strategies developed for the total synthesis of MCs.

**Figure 4:**
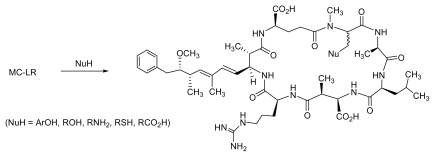
Conjugate addition of a wide range of nucleophiles to MC-LR.

Although the direct elaboration of the MC scaffold can offer immediate access to new analogues, its scope may be limited as the modification of the readily accessible functional groups (*i.e.*, Adda, D-Glu) may lead to compounds with a complete loss of cytotoxicity. Conjugate addition of glutathione (GSH) and cysteine (Cys) to *N*-methyldehydroalanine in MCs resulted in adducts with reduced toxicity, as seen upon assessing LD50 values using mice [145]. Considering that *N*-methyldehydroalanine may be responsible for the long-term hepatic retention and the toxicity of MCs, conjugate addition of a wide range of oxygen, nitrogen and sulfur based nucleophiles to the *N*-methyldehydroalanine double bond could give access to new MC analogs with reduced hepatic toxicity (Figure 4).

**Figure 5:**
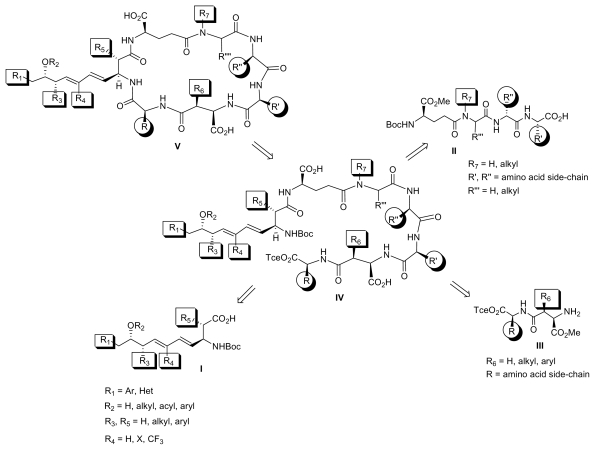
A combinatorial approach to structurally diverse microcystins.

This strategy would quickly clarify whether the dehydroalanine moiety is implicated in the long-term hepatic retention and the acute toxicity of the MC-LR. However, the limited availability of these toxins may hinder the scope of this strategy. Therefore, access to a series of structurally diverse new MC analogues with the desired pharmacological profile requires a combinatorial approach. We are currently in the process of preparing analogs with a wide range of diversity elements by solution or solid-phase strategies through the coupling of fragments I-III to tetrapeptide IV, which could in turn cyclize to MC variants with a general structure V (Figure 5).

### 6.2. Combinatorial Total Biosynthesis

Their macrocyclic ring enables MCs to adopt a high degree of structural pre-organization in such a way that critical functional groups can interact with their protein partners without any major entropic loss upon binding [136,146–149]. This pre-organized ring structure can lead to high affinity and selectivity for protein targets, preserving at the same time appropriate bioavailability. Despite the therapeutic potential of these compounds and the fact that more than 100 marketed macrocyclic drugs have been derived from natural products, cyanobacterial cyclopeptides have been ill-exploited for the discovery of novel anticancer compounds [132]. This under-exploration is due to challenges imposed by their structural complexity in their chemical synthesis during the lead optimization process. Therefore, novel strategies are required to effectively screen the sub-portion of the biologically active chemical space that could be sampled by novel MC analogues.

Microcystin biosynthesis is accomplished on large (55-kb) non-ribosomal peptide synthetase gene clusters (NRPS) [150,151] that include mixed peptide synthetases polyketide synthases, peptide synthetases, tailoring enzymes, and polyketide synthases [151–154]. Numerous toxins, siderophores and antimicrobial compounds are synthesized in NRPS enzyme complexes [155,156]. Interestingly, more than 70 structural variants of MCs can be synthesized from these enzyme complexes with modifications in the amino acid or the peptide backbone [157,158]. This wide variety of MC analogues raises two critical questions: why and how can Nature perform this efficient combinatorial chemistry *via* NRPS? A potential rationalization for the requisiteness of such chemical variability could be ascribed to the necessity of the MC-producing microorganisms to provide diverse chemical signals to decode complex modes of microbial interactions or for defense purposes against other organisms.

NRPS are organized in modular assembly lines in which each module, made of conserved catalytic domains, incorporates a given monomer unit into the growing chain for the biosynthesis of complex MCs. Each domain within these modules can be responsible for the adenylation, thioester formation, condensation of specific amino acids, amino acid modification (*i.e.*, oxidation, heterocyclization, formylation, epimerization) [155,156,159]. The pattern in which these catalytic domains are assembled within the multifunctional enzymes determines the order and the number of the residues to be incorporated in the final product [156]. Thus, recombination within this assembly line is one of the main mechanisms determining the diversification of NRPSs [158]. A recent study focusing on the microcystin synthetase gene cluster [160] has demonstrated that functional peptide synthetases are created in nature through the transfer of adenylation domains without the concomitant transfer of condensation domains. Currently, there is heightened interest in engineering non-ribosomal peptide synthetases toward the creation of novel bioactive peptides [156]. Engineering single amino acid changes in the putative substrate binding sites of adenylation domains of the NRPS gene clusters could provide synthetic diversification by tuning the type of amino acid that is recognized and activated by the adenylation domain [161,162]. Another way that the NRPS could be tailored to allow the biosynthesis of novel MC peptides is the construction of chimeric enzymes where single domains or intact modules are exchanged [163]. To achieve efficient reengineering of this assembly line towards the design of new bioactive molecules with improved therapeutic properties, prior knowledge of the module or domain structure and interactions is of critical importance [164–168]. Reconstitution of native and engineered biosynthetic pathways for MCs in model heterologous hosts (*Escherichia coli and Saccharomyces cerevisiae*) could provide an efficient way to surmount complex problems associated with total chemical synthesis [169–171]. Such combinatorial total biosynthesis using heterologous expression systems could accelerate the exploration of biosynthetic potential of microorganisms and enable the construction of an economical and efficient platform for mass production of MC analogues. The very recent constructive efforts are starting to shed light on an understanding of the architecture of the enzymatic assembly lines for MC production that have evolved over millions of years. This understanding at the biosynthetic level, and in synergy with chemistry, could greatly contribute to the increasing structural diversity of MCs and open new avenues for the rational design of novel MC analogues with improved cancer therapeutic properties.

### 6.3. Selectivity and Function

Cancer selectivity is essential for candidate compounds to be developed into successful anticancer drugs. We presume that a degree of selectivity might be achieved for MC analogues through lead optimization strategies that are directed towards the development of MC-antidote conjugates. We also consider that using approaches that target exploiting genetic and metabolic differences between cancer and normal cells can help develop cancer selective MC analogues.

Currently we are working on the conjugation of the ROS-scavenging agent *N*-Acetylcysteine (NAC) to microcystin-LR *via* its sulfhydryl unit on the intent to develop microcystin-NAC conjugates, which could preferentially damage OATP expressing cancer cells and spare healthy tissues. This strategy is supported by data showing that NAC works differentially in healthy and cancerous tissues. Characteristically, animal and human studies have shown that normal liver cells exposed to various hepatotoxic agents including microcystin (animal studies) can be rescued by NAC if given early [172–175], which is not the case for cancer cells [12,176].

A major biochemical feature of MC toxicity is the intracellular generation of reactive oxygen species (ROS) [40,177]. This biological effect could turn out to be a competitive advantage for MC analogues, when they are considered as potential cancer therapeutics, given that the elevation of intracellular ROS above a threshold level seem to constitute the biochemical basis of ROS-mediated cancer therapeutics [178]. It is worth noticing that cancer cells live in a state of increased basal oxidative stress, which makes them vulnerable to further ROS insults induced by exogenous agents [179–181] Therefore, microcystin analogues can selectively kill OATP expressing cancer cells without causing significant toxicity to normal cells, by exploiting the redox difference between normal and cancer cells. We consider that this approach may be improved by OATP inhibitors, as are rifampicin and cyclosporine-A, which at carefully selected dosing schedules could fine tune optimal redox modulation at target tissues [182,183]. Moreover, it is considered that MC analogues might even provide a possibility for radical therapeutic approach for cancer by disturbing the redox balance in OATP expressing cancer stem-like cells, which are thought to share features of normal stem cells and also exhibit malignant cell characteristics in redox regulation [178,184].

A third approach we are currently working on is scanning human solid tumors for *SLCO* mutations on the aim to develop MC analogues with selective affinity towards cancer-specific OATPs [94,185,186]. Finally throughout our drug development program we are using functional activity-based protein profiling approaches coupled with network pharmacology aiming to select the most potent analogues with minimal off-target in healthy tissues [187,188].

## 7. Conclusions

In the era of targeted cancer therapy, cyanotoxins comprise a rich source of natural cytotoxic compounds with a potential to target cancers expressing specific uptake transporters. Moreover, their structure offers opportunities for combinatorial engineering to enhance the therapeutic index and resolve organ-specific toxicity issues. Considering cyanobacterial cyclopeptides as potential novel targeted anticancer therapeutics, we focus on developing microcystin analogues optimized to efficiently target OATP-expressing metastatic cancers that are resistant to conventional chemotherapy.

## Figures and Tables

**Table 1:** SLCO expression at the protein (P) and mRNA (m) level in normal and cancerous human tissues. Each color represents a different gene family.

Approved gene symbol	Expression in human normal tissue	Expression in human tumor tissue
*SLCO1A2*	Liver (m; P), Brain (m), blood barrier (P), Kidney (m; P), Testis (m), Prostate (m), Breast (m), Retina (m)	Glioma (m;P); Bone tumors (m)
*SLCO1B1*	Liver (m; P), Mononuclear cells (m)	HCC (m; P); Colorectal Cancer (m)
*SLCO1B3*	Liver (m; P), Cervix (m), Mononuclear cells (m)	Colon Cancer (m; P); Breast cancer (P); Non Small Cell Lung Cancer (m); HCC (m; P)
*SLCO1C1*	Brain (m); Testis (m; P), Heart (m), Retina (m), Breast (m)	Glioma (m); Bone tumors (m)
*SLCO2A1*	Ubiquitous (protein detected only in GI tract tissue)	Colon cancer (m); Lung cancer (m); Bone tumor (m), Breast cancer (m)
*SLCO2B1*	Ubiquitous (protein detected only in liver tissue)	Glioma (m; P); Colon cancer (m); Lung cancer (m); Bone tumors (m); Breast cancer (m)
*SLCO3A1*	Ubiquitous and also in Peripheral Blood Mononuclear Cells (PBMC) (data available only on mRNA level)	Lung cancer (m); Colon cancer (m); Bone tumors (m); Breast cancer (m)
*SLCO4A1*	Ubiquitous (protein detected only in brain and placenta tissues)	Glioma (m); Lung cancer (m); colon cancer(m), Bone tumor (m); Breast cancer (m)
*SLCO4C1*	Kidney (m), Lung (m), Skin (m), PBMC (m), Kidney (m), Liver (m), Neutrophils (m), Breast (m), peripheral leukocytes (m)	Lung cancer (m); Bone tumor (m); Breast cancer (m)
*SLCO5A1*	Prostate (m), Skeletal muscles (m), Thymus (m), Classically activated macrophages (m), Breast (m).	Bone tumors (m); Breast cancer (m)
*SLCO6A1*	Testis (m), Spleen (m), Brain (m) (especially fetal brain), Placenta (m)	Non small cell lung cancer (m); Bladder cancer (m); Esophagus cancer (m); medulloblastoma (m)

**Table 2:** Comparison of IC50 (nM) values of synthetic MC-LA and variants in relation to the purified catalytic subunits of PP1 and PP2A.

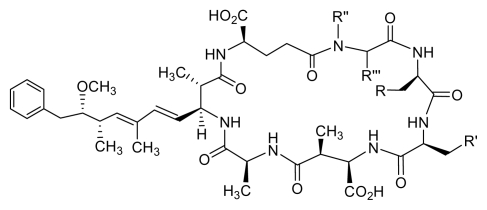							
					Inhibition (IC_50_ nm)		
Inhibitor	R	R′	R″	R‴	PP1c	PP2Ac	PP1 selectivity
MC-LA (synthetic)	H	CH(CH_3_)_2_	CH_3_	=CH_2_	0.3	0.3	1
**1**	H	Cyclohexyl	CH_3_	CH_2_	0.52	3.4	7
**2**	H	*i*-Propyl	Cyclohexyl	H	0.8	1.5	2
**3**	H	*i*-Propyl	CH_3_	H	0.8	1.5	2
**4**	NH_3_^+^	*i*-Propyl	CH_3_	CH_2_	3	9	3
